# Supplementary material for: Empirical methods for controlling false positives and estimating confidence in ChIP-Seq peaks
Source: BMC Bioinformatics. 2008 Dec 5;9:523. doi: 10.1186/1471-2105-9-523 (PMC2628906; doi:10.1186/1471-2105-9-523)
Supplement: Additional File 1 — A variety of html documents from the USeq web site detailing the available applications, their best usage, output file type descriptions, command line menus, etc. [file 1471-2105-9-523-S1.zip › USeqUserGuides/JavaDoc.zip!/JavaDoc/util/bio/annotation/class-use/StartStop.html]

Uses of Class util.bio.annotation.StartStop


|  |  |  |
| --- | --- | --- |
| **Overview** \| **Package** \| **Class** \| **Use** \| **Tree** \| **Deprecated** \| **Index** \| **Help** | |  |
| PREV   NEXT | **FRAMES**    **NO FRAMES**     **All Classes** |  |


---


## **Uses of Class util.bio.annotation.StartStop**

| Packages that use StartStop | |
| --- | --- |
| **util.bio.annotation** | For representing gene annotation. |
| **util.bio.seq** | Nucleic acid sequence manipulation. |

Uses of StartStop in util.bio.annotation

| Methods in util.bio.annotation with parameters of type StartStop | |
| --- | --- |
| `static int[]` | `StartStop.startsInBases(StartStop[] ss)`             Returns the starts in number of bases, not coordinates for the array. |
| `static int` | `StartStop.totalBP(StartStop[] ss)`             Returns the total number of bases, assumes interbase coordinates. |

Uses of StartStop in util.bio.seq

| Methods in util.bio.seq with parameters of type StartStop | |
| --- | --- |
| `java.util.ArrayList<java.lang.Integer>` | `OligoTiler.findCenters(StartStop ss)` |

---


|  |  |  |
| --- | --- | --- |
| **Overview** \| **Package** \| **Class** \| **Use** \| **Tree** \| **Deprecated** \| **Index** \| **Help** | |  |
| PREV   NEXT | **FRAMES**    **NO FRAMES**     **All Classes** |  |


---
